# A straightforward one-step strategy for SARS-CoV-2 diagnosis and screening of variants of concern: a multicentre study

**DOI:** 10.1590/0074-02760220202

**Published:** 2023-03-17

**Authors:** Marcela Fontana-Maurell, Fernando do Couto Motta, Monica Barcellos Arruda, Pedro Cardoso, Marisa Ribeiro, Elisabete Andrade, Daniela T Godoy, Elaine Costa, Daniele Rocha, Marilda Agudo MT Siqueira, Rodrigo Brindeiro, Patrícia Alvarez

**Affiliations:** 1 Fundação Oswaldo Cruz-Fiocruz, Instituto de Tecnologia de Imunobiológicos Bio-Manguinhos, Rio de Janeiro, RJ, Brasil; 2 Universidade Federal do Rio de Janeiro, Departamento de Genética, Rio de Janeiro, RJ, Brasil; 3 Fundação Oswaldo Cruz-Fiocruz, Instituto Oswaldo Cruz, Laboratório de Vírus Respiratório e Sarampo, Rio de Janeiro, RJ, Brasil

**Keywords:** SARS-CoV-2 diagnosis, COVID-19, variants of concern, epidemiological surveillance

## Abstract

**BACKGROUND:**

The prevalence of severe acute respiratory syndrome coronavirus 2 (SARS-CoV-2) variants of concern (VOCs) has changed unevenly over time around the world. Although whole genome sequencing is the gold standard for virus characterisation, the discovery of alpha VOC causing spike gene target failure (SGTF) result, when tested using an reverse transcription real-time polymerase chain reaction (RT-qPCR) assay, has provided a simple tool for tracking the frequencies of variants.

**OBJECTIVES:**

The aim of this study was to investigate if a multiplex RT-qPCR assay (BioM 4Plex VOC) could be used to detect SARS-CoV-2 and to perform a VOC screening test in a single reaction tube. Here, we present the multicentre study evaluating this assay.

**METHODS:**

Twelve laboratories have participated in the multicentre study. The BioM 4Plex VOC was distributed to them with detailed instructions of how to perform the test. They were asked to test the BioM 4Plex VOC in parallel with their routine Commercial SARS-CoV-2 diagnostic assay. Additionally, they were requested to select SARS-CoV-2-positive samples with genome sequenced and lineage definition according to PANGO lineage classification.

**FINDINGS:**

The BioM 4Plex VOC and commercial RT-PCR assay are equally effective in detecting SARS-CoV-2. Results revealed a specificity of 96.5-100% [95% confidence interval (CI)], a sensitivity of 99.8-100% (95% CI), and an accuracy of 99.8-100% (95% CI). A 99% concordance rate was found between results from the BioM 4Plex VOC and that from available genome sequencing data.

**MAIN CONCLUSIONS:**

The BioM 4Plex VOC provides an effective solution to detect SARS-CoV-2 infections and screening for VOCs in a single reaction. It is a straightforward method to help us monitor the frequency and distribution of VOCs and develop strategies to better cope with the pandemics.

In December 2019, China reported a cluster of cases of pneumonia of unknown cause detected in Wuhan City, Hubei Province.^1^ On January 5, 2020, the World Health Organization (WHO) published the first disease outbreak report on the new virus causing the pneumonia cases, and on January 11, 2020, China shared the genetic sequence of the new coronavirus, referred to as severe acute respiratory syndrome coronavirus 2 (SARS-CoV-2), the causative agent of coronavirus disease 2019 (COVID-19).^1^ ^,^ ^2^ Knowledge of the viral genome enabled the development of the first reverse transcription-polymerase chain reaction (RT-PCR) assay for virus detection.^2^ The disease has rapidly spread all over the world and on January 30, 2020, the WHO declared the COVID-19 outbreak a Public Health Emergency of International Concern and shortly after, on March 11, 2020, a global pandemic.^1^

In Brazil, the first COVID-19 case was confirmed on February 26, 2020.^3^ As of January 6, 2022, there were 22,184,824 reported cases and 616,691 deaths confirmed in Brazil according to the Ministry of Health.^4^

Genomic sequencing efforts have scaled massively during the COVID-19 pandemic with a large number of SARS-CoV-2 whole genome sequences generated all around the world. Although the number of genome sequences is remarkable, submissions are biased toward regions and countries with specialised genomic facilities with high sequencing capacity and groups with adequate and even access to reagents, sequencing platforms, and dedicated personnel to perform analysis.^5^

Mutations are a natural and expected part of the evolution process of viruses; the massive spread of SARS-CoV-2 across the world led to the rapid accumulation of mutations in the viral genome. The WHO has been monitoring those mutations through genomic surveillance and assessing the evolution of the virus. Since the emergence of SARS-CoV-2, more than 1,915 variants up to May 31, 2022, have been identified through viral genome sequencing (https://cov-lineages.org/lineage_list.html). During late 2020, the emergence of variants that posed an increased risk to global public health prompted the WHO to differentiate SARS-CoV-2 lineages into two categories, namely variants of interest (VOIs) and variants of concern (VOCs), to prioritise global monitoring and research.^6^ ^,^ ^7^ ^,^ ^8^ VOCs are SARS-CoV-2 variants that have been demonstrated to be associated with an increase in transmissibility, change in clinical disease presentation, or a decrease in the effectiveness of public health and social measures or available diagnostics, vaccines, or therapeutics.^7^

The currently designated VOCs are (i) Alpha, first documented in the United Kingdom, September 2020; (ii) Beta, South Africa, May 2020; (iii) Gamma, Brazil/Japan, January 2021; (iv) Delta, India, May 2021; and (v) Omicron, likely South Africa, November 2021.^7^ ^,^ ^9^

In Brazil, the temporal prevalence of circulating SARS-CoV-2 lineages was marked initially by the dominance of lineages B.1.1.28 and B.1.1.33^10^ ^,^ ^11^ ^,^ ^12^ followed by the spread of VOI Zeta (P2), VOC Gamma (P1),^12^ ^,^ ^13^ VOC Delta, and, more recently, VOC Omicron. According to the COVID-19 Fiocruz Genomic Surveillance Network, in January 2022, VOC Omicron accounts for more than 97% of sequenced genomes, replacing VOC Delta (2.5%) as the currently predominant lineage in Brazil (http://www.genomahcov.fiocruz.br/dashboard-en/).

The prevalence of variant detection changes over time and tracking such changes using genomic surveillance is difficult due to a myriad of technical and analytical constraints, from supply chain disruption to lack of personnel with expertise in genetic data curation and analysis, especially at poorly funded research institutes in developing countries. Still, the identification and relative distribution of VOCs remain of great importance for the implementation of local public health measures. RT-PCR assays can be easily developed and implemented to assist SARS-CoV-2 genomic surveillance.

The discovery of alpha VOC causing spike gene target failure (SGTF) result when tested using the TaqPath PCR assay provided labs throughout the world with a simple tool for tracking the frequencies of variants.^14^ ^,^ ^15^ ^,^ ^16^ The validated open-source PCR assay protocol, designed and published by Vogels and co-workers,^17^ targeting ORF1a Δ3675-3677 and spike Δ69-70, is another example of PCR tool that can be used to track VOCs. The aim of this study was to investigate if a multiplex reverse transcription real-time polymerase chain reaction (RT-qPCR) assay (SARS-CoV-2 4-Plex VOC Bio-Manguinhos RT-qPCR assay) could be used to detect SARS-CoV-2 and to perform a VOC screening test in a single reaction tube. Here, we present the multicentre study evaluating this assay.

## MATERIALS AND METHODS

*SARS-CoV-2 4-Plex VOC Bio-Manguinhos RT-qPCR assay (BioM 4Plex VOC)* - The SARS-CoV-2 4-Plex VOC Bio-Manguinhos molecular assay, named hereafter as BioM 4Plex VOC, is a RT-qPCR, with an internal control (IC), designed to detect SARS-CoV-2 infection and to perform a VOC screening test in a single reaction using nasopharyngeal swab samples that were previously subjected to nucleic acid extraction. Primers and probes target (i) the human constitutive gene RNase P as an internal control (ROX); (ii) SARS-CoV-2 E gene (VIC);^13^ (iii) the ORF1a Δ3675-3677 (Cy5), and (iv) spike Δ69-70 (FAM).^17^

All RT-qPCR assays were performed using 10 µL reaction mixtures of the QuantiNova Pathogen Kit (QIAGEN), 150 nM primers spike Δ69-70, 250 nM primers ORF1a Δ3675-3677, 250 nM primer E gene, 100 nM probe spike Δ69-70, 200 nM probe ORF1a Δ3675-3677, 250 nm probe E gene, and 10 µL nucleic acid eluate.

Target amplification was performed on an Applied Biosystems 7500 Real-Time PCR System (Thermo Fisher, Waltham, MA, USA) with the following conditions: reverse transcription at 50ºC for 15 min, initial denaturation 95ºC for 2 min, followed by 40 cycles at 95ºC for 20 s and 58ºC for 30 s. The QuantStudio™ Design and Analysis Software version 2.6 for QuantStudio™ 6/7 Pro Real-Time PCR systems (Thermo Fisher) was used to analyse RT-PCR data. A positive result was considered any amplification curve crossing the threshold value before cycle 35 for RNase P and cycle 40 for the other targets. A detailed interpretation of the amplification plot is provided in Table I.

**TABLE I:** Interpretation of results from the severe acute respiratory syndrome coronavirus 2 (SARS-CoV-2) 4-Plex variants of concern (VOC) Bio-Manguinhos reverse transcription real-time polymerase chain reaction (RT-qPCR) assay (BioM 4Pex VOC)

Result	RNase P	E gene	WT ORF1a Δ3675-3677	WT Δ69-70
Invalid test	Undetected	NA	NA	NA
SARS-CoV-2-negative	Detected	Undetected	Undetected	Undetected
SARS-CoV-2 positive, wild type to both deletions	Detected	Detected	Detected	Detected
SARS-CoV-2-positive, Spike Δ69/70 and ORF1a Δ3675-3677	Detected	Detected	Undetected	Undetected
SARS-CoV-2-positive, Spike Δ69/70	Detected	Detected	Detected	Undetected
SARS-CoV-2-positive, ORF1a Δ3675-3677	Detected	Detected	Undetected	Detected

Δ:deletion; NA: when RNase P is negative, the run is invalid; WT: wild-type “undeleted”.

*Study design* - Twelve laboratories in 11 Brazilian states agreed to participate in the study. For data analysis, each laboratory was randomly assigned a code number. The list of participating laboratories is presented at the end of the text, with the collaborative study group details.

The BioM 4Plex VOC was distributed to all participating laboratories (listed in the collaborative study group details) together with detailed instructions of how to perform the test. In each laboratory, viral RNA was extracted from nasopharyngeal swabs according to the extraction kit manufacturer’s instructions. Ten microliters of RNA were used for the BioM 4Plex VOC. Participating laboratories tested the BioM 4Plex VOC in parallel with their routine SARS-CoV-2 diagnostic assay or used SARS-CoV-2-positive samples available in their facilities. The results were expressed as cycle threshold (Ct) values for each target and assay.

Participating laboratories were requested to select as many SARS-CoV-2-positive samples with genome sequenced and lineage definition according to PANGO lineage classification^18^ as possible. To perform a comprehensive paired sample analysis, laboratories were also asked to provide the Ct value obtained with the protocol originally used for SARS-CoV-2 detection. Those data were used to clinical evaluation, such as, sensitivity, specificity, and accuracy.

*Analytical evaluation* - The BioM 4Plex VOC limit of detection (LoD) and linearity was determined using the AccuPlex™ SARS-CoV-2 Verification Panel - Full Genome (material no. 0505-0168; SeraCare, Milford, MA, USA). This positive reference material contains recombinant virus particles with sequences containing the entire SARS-CoV-2 genome (GenBank accession number NC_045512.2). The standard used had 1,000 copies/µL and was tested in a two-fold dilution with eight replicates each. The results were used to calculate the 95% LoD with a PROBIT analysis (IBM SPSS Statistics Subscription).

The Laboratory for Respiratory Viruses and Measles, from Oswaldo Cruz Foundation (FIOCRUZ), is the national reference for respiratory viruses and has provided for this study, results from testing the BioM 4Plex VOC with a panel of respiratory viruses, formed by the following viruses: Influenza (A and B), respiratory syncytial virus (RSV), rhinovirus, adenovirus, metapneumovirus (hMPV) and measles. Those data were also used for clinical evaluation, such as sensitivity, specificity, and accuracy.

*Statistical methods* - Comparison between paired samples of Ct values for virus detection were made with GraphPad Prism (version 9.2.1) and test T-student was used considering normal distribution. Linear regression analysis was performed for graphic and numeric analysis of the correlation between methods. Pearson correlation was performed for linearity analysis. Statistical analyses were performed using XLSTAT 2020.5.1 (Addinsoft, New York, USA). Sensitivity, specificity, and accuracy were calculated with MedCalc Software Ltd. Diagnostic test evaluation calculator (Version 20.110).^19^

## RESULTS

*BioM 4Plex VOC and commercial RT-PCR assay are equally effective in detecting SARS-CoV-2* - Our multicentre study had the participation of 12 laboratories. Eleven participating laboratories from 11 Brazilian states provided SARS-CoV-2 testing data. One laboratory has contributed with a panel of 95 true negative SARS-CoV-2 samples from blood blanks. Blood banks often send surpluses of negative plasma to Bio-Manguinhos (Fiocruz). Those samples were subjected to comprehensive serological and molecular characterisation prior to inclusion in an internal reference panel and were treated as true negative samples in this study.

Eleven laboratories provided results of Ct values from 2,758 positive samples tested with the BioM 4Plex VOC. Participating laboratories were also asked to submit Ct results from routine RT-PCR assays. Any RT-PCR assay commercially available in Brazil and approved by Brazilian regulatory agency ANVISA could be used. Additionally, laboratories were asked to not report the type of test used, only the Ct value for E/N targets. Of the 2,758 positive samples, 2,622 had Ct values for E/N targets from both assays, a commercial RT-PCR assay (mean Ct 20.31 ± 4.10) and the BioM 4Plex VOC (mean Ct 19.22 ± 4.24). From 136 samples we have received only Ct values from BioM 4Plex VOC.

**Fig. 1:**
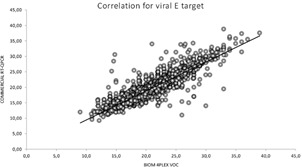
linear regression of cycle threshold (Ct) values for the severe acute respiratory syndrome coronavirus 2 (SARS-CoV-2) 4-Plex variants of concern (VOC) Bio-Manguinhos reverse transcription real-time polymerase chain reaction (RT-qPCR) assay (BioM 4Pex VOC) and commercial E/N RT-qPCR assays (R^2^ = 0.8264).

**Fig. 2:**
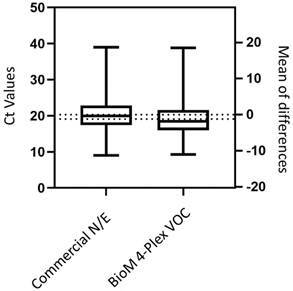
box plot of mean values and interquartile range for total paired samples targeting the E gene. The severe acute respiratory syndrome coronavirus 2 (SARS-CoV-2) 4-Plex variants of concern (VOC) Bio-Manguinhos reverse transcription real-time polymerase chain reaction (RT-qPCR) assay (BioM 4-Plex VOC) is slightly more sensitive than commercial N/E (Paired T test, p < 0.001).

In total, 2,853 samples (2,758 positive and 95 negative samples for SARS-CoV-2) from 12 laboratories were analysed using the BioM 4Plex VOC. Table II shows the number of samples tested in each participating laboratory and the mean Ct values from SARS-CoV-2 detection for commercial RT-PCR assays and the BioM 4Plex VOC. Fig. 1 shows the linear regression regarding the correlation of Ct values from BioM 4-Plex VOC targeting the E gene and the commercial E/N RT-PCR assay (R² = 0.8264). Fig. 2 shows the box plot of mean values for 2622 paired samples. The mean Ct for BioM 4Plex VOC is slightly smaller than commercial N/E and (p < 0.001). Pearson correlation coefficient was -0.8.

**TABLE II:** Mean cycle threshold (Ct) values for commercial coronavirus disease 2019 (COVID-19) reverse transcription-polymerase chain reaction (RT-PCR) assays and the severe acute respiratory syndrome coronavirus 2 (SARS-CoV-2) 4-Plex variants of concern (VOC) Bio-Manguinhos reverse transcription real-time polymerase chain reaction (RT-qPCR) assay (BioM 4Pex VOC)

Laboratory	N	Commercial N/E Mean Ct (SD)	BioM 4-Plex VOC Mean Ct (SD)
1	95		
2	36		22.69 (± 3.71)
3	57	21.8 [n = 43]^*^ (± 4.61)	23.76 (± 6.32)
4	188	21.68 [n = 102]^*^ (± 4.56)	21.21 (± 4.15)
5	129	20.37 (± 3.49)	19.19 (± 3.43)
6	328	21.16 (± 4.30)	20.12 (± 4.17)
7	641	18.83 (± 3.46)	17.6 (± 3.39)
8	93	19.71 (± 3.88)	19.28 (± 4.31)
9	320	20.38 (± 3.21)	18.98 (± 3.29)
10	86	25.44 (± 6.72)	22.18 (± 6.16)
11	94	18.61 (± 3.53)	17.57 (± 3.58)
12	786	20.57 (± 3.82)	18.98 (± 3.94)
Total	2,853	20.31 (± 4.10)	19.22 (± 4.24)

*The number of samples tested with the commercial assay (in brackets) is different from the BioM 4-Plex VOC assay. SD: standard deviation

*Analytical results from BioM 4Plex VOC* - Using the AccuPlex™ SARS-CoV-2 Verification Panel, the 95% Limit of Detection (LOD) was calculated to be 6.9 copies/reaction for the E gene, 4.2 copies/reaction for the spike gene, and 7.4 copies/reaction for ORF1a gene. The linear range of the assay extended from 185 copies/ reaction to 1.44 copies/reaction.

To check the specificity of BioM 4Plex VOC, 95 truly negative samples from blood banks were tested with no false positive signal. Additionally, a panel of respiratory viruses were also tested, with no false positive signal when testing Influenza (A and B), respiratory syncytial virus (RSC), rhinovirus, adenovirus, metapneumovirus (hMPV) and measles. The analysis of 102 truly negative samples and 1,568 positive samples (with sequencing data) revealed a specificity of 100% (96.5-100%; 95% CI), a sensitivity of 100% (99.8-100%; 95% CI), and an accuracy of 100% (99.8-100%; 95% CI).

**Fig. 3:**
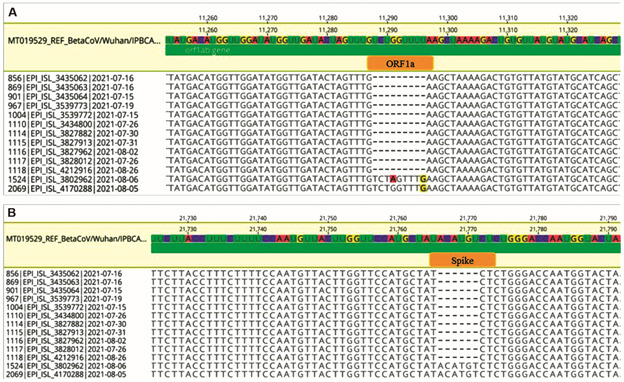
sections of alignment from 13 samples presenting discordant severe acute respiratory syndrome coronavirus 2 (SARS-CoV-2) 4-Plex variants of concern (VOC) Bio-Manguinhos reverse transcription real-time polymerase chain reaction (RT-qPCR) assay (BioM 4-Plex VOC) results in comparison to whole genome sequencing (WGS). The first eleven samples presented deletions in both ORF1a (A) and Spike (B) genes. Despite this pattern being characteristic of Alpha lineage, these samples are exceptionally classified as Gamma by whole-genome analysis. On the other hand, the last two samples (1524 and 2069) were genetically characterised as Gamma samples despite the absence of searched deletions in both regions. All genomes are available at GISAID under the accession codes EPI_ISL_3435062, EPI_ISL_3435063, EPI_ISL_3435064, EPI_ISL_3539773, EPI_ISL_3539772, EPI_ISL_3434800, EPI_ISL_3827882, EPI_ISL_3827913, EPI_ISL_3827962, EPI_ISL_3828012, EPI_ISL_4212916, EPI_ISL_3802962, EPI_ISL_4170288 (Supplementary data).

*BioM 4Plex VOC can be used to screen VOCs* - The concordance of the BioM 4Plex VOC and the gold standard whole genome sequencing (WGS) grouped by participating laboratory is shown in detail in Table IV. The comparison of the 1,568 SARS-CoV-2 samples with available genome sequencing data and results from the BioM 4Plex VOC revealed 1,555 concordant samples and only 13 discordant samples. Eleven samples were identified as VOC Alpha, with deletions to both *ORF1a* and *Spike*, but WGS results identified them as lineages P.1. Two samples were identified VOC Gamma, with deletion to *ORF1a* target only, but WGS results identified them as VOC Delta. Table III shows the mutation profile for each VOC. The alignment, with GISAID accession number, from those 13 samples presenting discordant results are presented in Fig. 3.

**TABLE III:** Profile of variants of concern (VOCs) with spike and ORF1a targets

VOC	First detected	Spike 69/70	ORF1a
Alpha	United Kingdom	-	-
Omicron	South Africa	-	-
Beta	South Africa	+	-
Gamma	Brazil	+	-
Delta	India	+	+

**TABLE IV:** Concordance of the severe acute respiratory syndrome coronavirus 2 (SARS-CoV-2) 4-Plex variants of concern (VOC) Bio-Manguinhos reverse transcription real-time polymerase chain reaction (RT-qPCR) assay (BioM 4-Plex VOC) and whole genome sequencing (WGS)

Laboratory	1^*^	2	3	4	5	6	7	8	9	10	11	12	Total
Total samples	95	36	57	188	129	328	641	93	320	86	94	786	2,853
Samples with WGS	0	0	33	102	112	270	126	85	2	0	94	744	1,568
Concordant with WGS	‒	‒	33	102	112	270	115	84	2	‒	94	743	1,555
Discordant with WGS	‒	‒	0	0	0	0	11	1	0	‒	0	1	13
% concordance	‒	‒	100.0%	100.0%	100.0%	100.0%	90.5%	98.8%	100.0%	‒	100.0%	99.9%	99.0%

*All samples tested in this laboratory were true negative SARS-CoV-2 samples.

## DISCUSSION

The BioM 4Plex VOC provides a solution to detecting SARS-CoV-2 infections and screening for VOCs in a single reaction based on the presence or absence of specific set of deletions (spike Δ69-70 and or ORF1a Δ3675-3677). The results obtained with both target genes allows for the screening of VOCs Alpha/Omicron, Beta/Gamma, and Delta with great concordance with WGS.

Twelve laboratories from different regions in Brazil participated in this multicentre study evaluating BioM 4Plex VOC and providing results from previously tested samples that were confirmed to be positive or negative for SARS-CoV-2. Whenever possible, participating laboratories were asked to select samples for which complete genome sequencing data were available. All positive SARS-CoV-2 samples, independent of virus lineage, tested with the BioM 4Plex VOC were accurately detected, indicating that this novel diagnostic test is an effective solution to SARS-CoV-2 detection.

Efficient diagnostic testing is an important tool for pandemic management and control. The performance of BioM 4Plex VOC was equivalent to the commercial COVID-19 RT-PCR assays as presented in Table II and Fig. 2. Additionally, BioM 4Plex VOC has shown to be sensitive to detect SARS-CoV-2, with LOD of 6.9 copies/reaction for E gene. The sensitivity of RT-qPCR assays, published previously, ranges from 3.8 to 10 RNA copies per reaction.^20^ The WHO proposed a set of criteria named ASSURED (Affordable, Sensitive, Specific, User-friendly, Rapid and robust, Equipment-free, and Deliverable to end-users) that could be used to determine if the diagnostic method meet the demands of epidemic control.^21^ However, few diagnostic methods meet all criteria. The BioM 4Plex VOC is sensitive (99.8-100%), specific (96.5-100%) and accurate (99.8-100%). Although not equipment-free, BioM 4Plex VOC uses a robust platform of equipment and trained professionals already implemented in Brazilian public healthcare network.

A SARS-CoV-2 variant-specific RT-qPCR assay provides a great contribution to assist with genomic surveillance and represents an effective tool for enabling a more equitable global response to emerging SARS-CoV-2 variants.^14^ ^,^ ^22^ The BioM 4Plex VOC is a highly sensitive, cost-effective multiplex assay that enables rapid detection of SARS-CoV-2 for the diagnosis and screening of VOCs circulating in Brazil. A similar strategy using SARS-CoV-2 variant-specific RT-PCR assays has been successfully employed in the Netherlands and France.^16^ ^,^ ^22^ In France, PCR-based screening for SARS-CoV-2 variants with spike deletion ΔH69/ΔV70 allowed for the first detection of VOC Alpha (previously known as 202012/01) in the country.^16^

Brazil has continental dimensions and, as such, inequalities exist in the distribution of research facilities and resources between regions. As of January 11, 2022, the COVID-19 Fiocruz Genomic Surveillance Network (http://www.genomahcov.fiocruz.br/dashboard-en/) has sequenced 94,188 complete SARS-CoV-2 genomes, which accounts for 0.4% of all SARS-CoV-2-positive cases in Brazil. According to Oude Munnink and colleagues,^5^ as of July 5 2021, 37,913 whole genome sequences from South America (0.12% of all reported SARS-CoV-2-positive cases from that continent), 692,704 from North America (1.75% coverage), 1,292,415 from Europe (2.35% coverage), 25,284 from Africa (0.32% coverage), 146,562 from Asia (0.3% coverage), and 20,613 from Oceania (25.0% coverage) had been generated.

WGS is the gold standard for identifying new variants. However, it is time-consuming and expensive compared to real-time RT-PCR. Therefore, not every sample detected is sequenced. Currently, the choice of samples to be sequenced is based on three parameters: the samples must have a Ct value less than 28 (the cut-off factor), as it increases the chance of a successful sequencing; the region where the sample was collected and the moment of collection. Finally, a feasible amount of samples is randomly picked from this list. Despite this strategy that looks at optimise sampling in time and space, the chances of early detection of merging variants. The BioM 4Plex VOC is a one-step strategy for SARS-CoV-2 detection and mutation screening in VOCs circulating in Brazil, improving the epidemiological surveillance. The assay can be used to prioritise samples for sequencing and to help monitor the distribution of confirmed and suspected variants, as well as detect new variants that do not match the pattern found in any of these variants.

A 99% concordance rate was found between results from the BioM 4Plex VOC and that from available genome sequencing data. Reliable genotyping of SARS-CoV-2 variants can be of great value and an alternative tool that complements the genomic sequencing surveillance system. In fact, variant-specific RT-qPCR screening has been shown to be a viable approach when resources for genomic surveillance are limited due to lack of funding and/or expertise,^16^ ^,^ ^22^ and even in regions with straightforward access to whole genome sequencing facilities, sequencing may be limited due to low viral loads.^14^ Moreover, sequencing facilities are not readily accessible for the vast majority of cities in Brazil and the turnaround time of genomic sequencing is relatively long. In contrast, every state in Brazil has a central laboratory with an RT-qPCR capability and expertise. Thus, the BioM 4Plex VOC can be extensively used across the country, enhancing variant surveillance and detection of SARS-CoV-2.

We recognise that our new diagnostic assay has some limitations. The BioM 4Plex VOC cannot identify VOCs that do not carry genomic signatures within a specific set of deletions (spike Δ69-70 and/or ORF1a Δ3675-3677). Nevertheless, if a new VOC is identified, a new pair of variant-specific primers and probe can be easily designed, and an updated RT-qPCR assay can be developed and implemented shortly. Another limitation of our assay is that it cannot detect sublineages and samples with atypical variations in the target genes. Based on WGS analysis, eleven discordant samples were classified as P.1, all Gamma. Those samples present ORF1a and Spike deletions, which made them erroneously classified as Alpha by BioM 4Plex VOC. Noteworthy, BioM 4Plex VOC has worked perfectly, since those samples present both deletions. The limitation demonstrated here is that although the great majority of samples can be correctly classified by the RT-qPCR assay, some exception subgroups can be misclassified by using these two deletions alone. In addition, is important to keep in mind that new lineages constantly evolve, making necessary a constant evaluation of these two deletion patterns. Recently, we started using this protocol to make the differentiation between residual Delta samples and Omicron, as well as between omicron sub-lineages BA.1, BA.3, and BA.5 from BA.2 and BA.2.12.1. The last two disagreed sequences are from other P.1 sub-lineages and do not present any of the two deletions investigated. These sub-groups are also represented in small numbers overall. These results show that continuous follow up of a representative set of RT-PCR-screened samples using WGS is crucial to identify these possibly rare variants that may emerge.

The BioM 4Plex VOC successfully detected all SARS-CoV-2-positive samples with or without deletions in the spike and/or nsp6 gene, and this is another attribute of our assay that must be highlighted. Some RT-PCR assays were adversely affected by the emergence of VOC Alpha in the United Kingdom, leading to what has been termed SGTF or spike gene drop out.^14^ In Portugal, SGTF and spike gene late detection (SPTL) data were successfully used as a useful surrogate to track the spread of VOC Alpha (also known as B.1.1.7).^23^ However, a SGTF result is not definitive for VOC Alpha and the RT-PCR assay used at the time could not detect other VOCs that lack *Spike* ΔH69/ΔV70.^16^ More recently, preliminary evidence published by the WHO indicates that SGTF can also be used as a marker for the fast-spreading VOC Omicron.^8^ It is worth mentioning that SGTF means that one out of three targets from a diagnostic assay is not detected, which further highlights the importance of target selection in a molecular assay. In fact, every molecular assay targeting the spike gene has been affected by the emergence of SARS-CoV-2 variants with novel spike mutations.

Since the emergence of VOC Omicron, over 150,000 BioM 4Plex VOC reactions have been distributed to 27 Public State Laboratories in Brazil via the Ministry of Health and are being used routinely for detection and diagnosis of SARS-CoV-2 and screening of VOCs in a single reaction. Numerous Omicron samples have been detected at this time and the lineage has been confirmed by whole genome sequencing (data no shown).

As every other RT-PCR-based assay, the BioM 4Plex VOC can be easily adjusted to other emerging variants or pathogens and is perfectly suitable for monitoring VOCs. Importantly, although the BioM 4Plex VOC is already registered by the Brazilian regulatory agency - ANVISA, and is being used throughout the country, whenever necessary, the R&D team will be able to make any adjustments aiming to improve the assay.

In conclusion, our results show that the SARS-CoV-2 4-Plex VOC Bio-Manguinhos RT-qPCR assay is an effective tool for detection of SARS-CoV-2 infection and screening of VOCs in a single reaction tube. As the pandemic evolves, the importance of rapid, large-scale screening of SARS-CoV-2 variants becomes increasingly more evident, leading to an urgent need to develop more cost-effective and straightforward methods to help us monitor the surge of new variants and develop strategies to better cope with the pandemics.
