# ERRATUM

**DOI:** 10.1590/0074-02760230003ER

**Published:** 2023-04-21

In the article “**A straightforward one-step strategy for SARS-CoV-2 diagnosis and screening of variants of concern: a multicentre study**”, DOI number: 10.1590/0074-02760220202, published in *Mem Inst Oswaldo Cruz*, Rio de Janeiro, Vol. 118: e220202, 2023, on page 1:

Where it reads:

Marcela Fontana-Maurell^1,2^, Fernando do Couto Motta^3^, Monica Barcellos Arruda^1^, Pedro Cardoso^1^, Marisa Ribeiro^1^, Elisabete Andrade^1^, Daniela T Godoy^1^, Elaine Costa^1^, Daniele Rocha^1^, Marilda Agudo MT Siqueira^3^, Rodrigo Brindeiro^1,2^, Patrícia Alvarez^1^/^+^, The collaborative study group*

^1^Fundação Oswaldo Cruz-Fiocruz, Instituto de Tecnologia de Imunobiológicos Bio-Manguinhos, Rio de Janeiro, RJ, Brasil

^2^Universidade Federal do Rio de Janeiro, Departamento de Genética, Rio de Janeiro, RJ, Brasil

^3^Fundação Oswaldo Cruz-Fiocruz, Instituto Oswaldo Cruz, Laboratório de Vírus Respiratório e Sarampo, Rio de Janeiro, RJ, Brasil

It should read:

Marcela Fontana-Maurell^1,2^, Fernando do Couto Motta^3^, Monica Barcellos Arruda^1^, Pedro Cardoso^1^, Marisa Ribeiro^1^, Elisabete Andrade^1^, Daniela T Godoy^1^, Elaine Costa^1^, Daniele Rocha^1^, Paola Cristina Resende^3^, Marilda Agudo MT Siqueira^3^, Rodrigo Brindeiro^1,2^, Patrícia Alvarez^1^/^+^, The collaborative study group*

^1^Fundação Oswaldo Cruz-Fiocruz, Instituto de Tecnologia de Imunobiológicos Bio-Manguinhos, Rio de Janeiro, RJ, Brasil

^2^Universidade Federal do Rio de Janeiro, Departamento de Genética, Rio de Janeiro, RJ, Brasil

^3^Fundação Oswaldo Cruz-Fiocruz, Instituto Oswaldo Cruz, Laboratório de Vírus Respiratório e Sarampo, Rio de Janeiro, RJ, Brasil

